# Supplementary material for: Metabarcoding is (usually) more cost effective than seining or qPCR for detecting tidewater gobies and other estuarine fishes
Source: PeerJ. 2024 Feb 26;12:e16847. doi: 10.7717/peerj.16847 (PMC10903359; doi:10.7717/peerj.16847)
Supplement: Supplemental Information 1 [file peerj-12-16847-s001.docx]

**Table S1.** Tidewater goby detection parameters estimated from logistic regression for qPCR and metabarcoding at two sites where tidewater goby were known to occur.

|  | **Estimate** | **Std. Error** | **z value** | **Pr(>\|z\|)** |
| --- | --- | --- | --- | --- |
| (Intercept) | 5.0916139 | 3.634299 | 1.4009891 | 0.1612173 |
| relevel(factor(Method), ref = “qPCR”)goby.metabarcode | -82.0811460 | 21304.738181 | -0.0038527 | 0.9969260 |
| estuaryJ Street Canal:sample | 0.7511434 | 255.790376 | 0.0029366 | 0.9976570 |
| estuarySanta Clara River Mouth:sample | -0.2099705 | 0.272028 | -0.7718711 | 0.4401908 |
