# Supplementary material for: Metabarcoding is (usually) more cost effective than seining or qPCR for detecting tidewater gobies and other estuarine fishes
Source: PeerJ. 2024 Feb 26;12:e16847. doi: 10.7717/peerj.16847 (PMC10903359; doi:10.7717/peerj.16847)
Supplement: Supplemental Information 2 [file peerj-12-16847-s002.docx]

**Table S2.** Tidewater goby detection parameters estimated from logistic regression for seine and qPCR for several well sampled sites where tidewater gobies occur (Schmelzle and Kinziger (2015)).

|  | **Estimate** | **Std. Error** | **z value** | **Pr(>\|z\|)** |
| --- | --- | --- | --- | --- |
| (Intercept) | 1.0807385 | 0.4324914 | 2.4988672 | 0.0124591 |
| relevel(factor(Method), ref = “goby.seine”)qPCR | 1.5884599 | 0.2935705 | 5.4108286 | 0.0000001 |
| estuaryDavis_Lake | -1.2819961 | 0.7005242 | -1.8300524 | 0.0672421 |
| estuaryEel_River_Estuary_Preserve | 0.8615528 | 0.8615794 | 0.9999692 | 0.3173254 |
| estuaryHBNWR_SCU_North | -1.2259270 | 0.6396322 | -1.9166123 | 0.0552872 |
| estuaryHBNWR_SCU_South | -1.5822316 | 0.6933012 | -2.2821704 | 0.0224793 |
| estuaryLake_Earl | -1.3497925 | 0.5648658 | -2.3895809 | 0.0168676 |
| estuaryMartin_Slough_ | -0.4470204 | 0.7315460 | -0.6110626 | 0.5411581 |
| estuaryOcean_Ranch | -2.1873991 | 0.5901236 | -3.7066795 | 0.0002100 |
| estuarySalt_River | -0.4470204 | 0.7315460 | -0.6110626 | 0.5411581 |
| estuaryStone_Lagoon | -1.2496055 | 0.5601040 | -2.2310239 | 0.0256795 |
| estuaryTen_Mile_River | -1.3497925 | 0.6737431 | -2.0034231 | 0.0451319 |
| estuaryTillas_Slough | 0.0744040 | 0.7748895 | 0.0960189 | 0.9235056 |
