# Supplementary material for: Metabarcoding is (usually) more cost effective than seining or qPCR for detecting tidewater gobies and other estuarine fishes
Source: PeerJ. 2024 Feb 26;12:e16847. doi: 10.7717/peerj.16847 (PMC10903359; doi:10.7717/peerj.16847)
Supplement: Supplemental Information 3 [file peerj-12-16847-s003.docx]

**Table S3.** Detection by method at a low detection site (Ocean Ranch) Schmelzle and Kinziger (2015). gammaMean is the probability estimated from the logistic coefficient using linkinv function in fit. gammaUpper and gammaLower are the upper and lower 95% confidence intervals for pMean.

| **Method** | **gammaMean** | **gammaUpper** | **gammaLower** |
| --- | --- | --- | --- |
| goby.metabarcode | 0.6181727 | 0.7859262 | 0.4165522 |
| qPCR | 0.6181727 | 0.7859262 | 0.4165522 |
| goby.seine | 0.2484940 | 0.4342146 | 0.1247012 |
