# Supplementary material for: Metabarcoding is (usually) more cost effective than seining or qPCR for detecting tidewater gobies and other estuarine fishes
Source: PeerJ. 2024 Feb 26;12:e16847. doi: 10.7717/peerj.16847 (PMC10903359; doi:10.7717/peerj.16847)
Supplement: Supplemental Information 4 [file peerj-12-16847-s004.docx]

**Table S4.** Model fits for one sample per site for comparison with S&K.

|  | **Estimate** | **Std. Error** | **t value** | **Pr(>\|t\|)** |
| --- | --- | --- | --- | --- |
| z | -0.2461471 | 0.0295687 | -8.324588 | 0 |
| a | -1.5236350 | 0.0468221 | -32.540963 | 0 |
| b | -0.0840551 | 0.0077286 | -10.875859 | 0 |
