# Supplementary material for: Metabarcoding is (usually) more cost effective than seining or qPCR for detecting tidewater gobies and other estuarine fishes
Source: PeerJ. 2024 Feb 26;12:e16847. doi: 10.7717/peerj.16847 (PMC10903359; doi:10.7717/peerj.16847)
Supplement: Supplemental Information 5 [file peerj-12-16847-s005.docx]

**Table S5.** Model fits for two samples per site (as was done in my sampling)

|  | **Estimate** | **Std. Error** | **t value** | **Pr(>\|t\|)** |
| --- | --- | --- | --- | --- |
| z | -0.2901178 | 0.0201575 | -14.39252 | 0 |
| b | -1.1230032 | 0.0062592 | -179.41701 | 0 |
| c | 0.0138747 | 0.0007182 | 19.31902 | 0 |
| a | -2.1317342 | 0.0566730 | -37.61466 | 0 |
